# Structural basis of mismatch recognition by a SARS-CoV-2 proofreading enzyme

**DOI:** 10.1126/science.abi9310

**Published:** 2021-09-03

**Authors:** Chang Liu, Wei Shi, Scott T. Becker, David G. Schatz, Bin Liu, Yang Yang

**Affiliations:** 1 Department of Immunobiology, Yale School of Medicine, New Haven, CT, USA.; 2 Section of Transcription and Gene Regulation, The Hormel Institute, University of Minnesota, Austin, MN, USA.; 3 Roy J. Carver Department of Biochemistry, Biophysics and Molecular Biology, Iowa State University, Ames, IA, USA.

## Abstract

Coronavirus 3′-to-5′ exoribonuclease (ExoN), residing in the nonstructural protein (nsp) 10–nsp14 complex, boosts replication fidelity by proofreading RNA synthesis and is critical for the virus life cycle. ExoN also recognizes and excises nucleotide analog inhibitors incorporated into the nascent RNA, undermining the effectiveness of nucleotide analog–based antivirals. Here we present cryo–electron microscopy structures of both wild-type and mutant severe acute respiratory syndrome coronavirus 2 (SARS-CoV-2) nsp10-nsp14 in complex with an RNA substrate bearing a 3′-end mismatch at resolutions ranging from 2.5 to 3.9 angstroms. The structures reveal the molecular determinants of ExoN substrate specificity and offer insight into the molecular mechanisms of mismatch correction during coronavirus RNA synthesis. Our findings provide guidance for rational design of improved anticoronavirus therapies.

## A proofreader in SARS-CoV-2

Although vaccines provide protection against severe acute respiratory syndrome coronavirus 2 (SARS-CoV-2), there remains a need for antivirals to treat COVID-19. Nucleotide analog drugs such as remdesivir, which target the viral RNA polymerase, have potential but are compromised by exoribonuclease (ExoN) activity that removes incorrect nucleotides from newly synthesized RNA. Liu *et al*. determined the structure of the complex that harbors the ExoN activity (nsp10–nsp-14) bound to a mimic of RNA that has incorporated an incorrect nucleotide. The structure shows how the RNA is recognized and suggests how ExoN specifically removes mismatched nucleotides. It also provides clues for designing nucleotide analogs that may evade excision. —VV

## Teaser

Structures of SARS-CoV-2 exonuclease–RNA complex provide insight into proofreading for potential antiviral development.

Severe acute respiratory syndrome coronavirus 2 (SARS-CoV-2), the causative agent of the COVID-19 pandemic, has infected more than 160 million people and led to more than 3 million deaths worldwide (https://covid19.who.int). Although several SARS-CoV-2 vaccines are now available (*1*), there are no highly effective antiviral agents to treat the disease. One of the most important druggable targets for SARS-CoV-2 is its replication-and-transcription complex (RTC), a multisubunit machine that carries out viral genome replication and transcription and plays an essential role in the virus life cycle (*2*, *3*). Central to the coronavirus RTC is the core RNA-dependent RNA polymerase (RdRp); nonstructural protein 12 (nsp12) (*4*); and two associated accessory proteins, nsp7 and nsp8 (*5*). SARS-CoV-2 RdRp is a promising target for nucleotide analog antivirals such as remdesivir (*6*, *7*). However, the efficacy of nucleotide analog inhibitors on coronavirus RdRp is compromised by the presence of the viral nsp14 exoribonuclease (ExoN) (*8*, *9*), an RNA proofreader that is specific to coronaviruses and a few other closely related virus families of the Nidovirales order and crucial to maintain the integrity of their unusually large RNA genome (*9*–*11*). In addition, ExoNs from coronaviruses and other RNA viruses play a key role in the evasion of host immune responses by degrading the viral double-stranded RNA (dsRNA) intermediates that would otherwise be recognized by host pathogen recognition receptors (*12*–*15*).

**Fig. 1.**
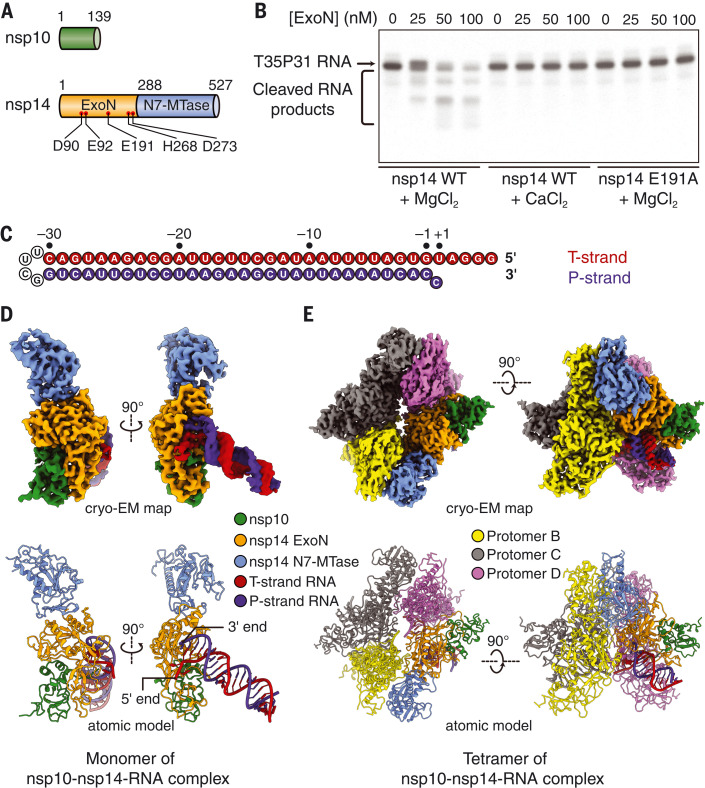
Structural and functional overview of SARS-CoV-2 nsp10-nsp14-RNA complexes. (**A**) Domain organization of SARS-CoV-2 nsp10 and nsp14. Domain boundary residues are numbered. Five catalytic residues in the nsp14 ExoN domain are indicated as red dots and are labeled. (**B**) Cleavage of T35P31 RNA substrate by SARS-CoV-2 nsp10-nsp14 ExoN complex. The nanomolar concentrations of WT or E191A mutant ExoN are indicated. The RNAs were resolved by denaturing polyacrylamide gel electrophoresis (PAGE) and stained by SYBR Gold. A representative result from three biological replicates is shown. (**C**) Sequence and numbering of the T35P31 RNA substrate used in biochemical characterization and structural determination. T-strand (template strand) and P-strand (product strand) RNAs are connected by a UUCG tetraloop. (**D**) Cryo-EM map and atomic model of the monomeric form of the SARS-CoV-2 nsp10-nsp14-RNA complex. (**E**) Cryo-EM map and atomic model of the tetrameric form of the SARS-CoV-2 nsp10-nsp14-RNA complex. A, Ala; D, Asp; E, Glu; H, His.

Nsp14 is a bifunctional enzyme that harbors both 3′-to-5′ ExoN and mRNA cap guanine-N7 methyltransferase (N7-MTase) activities (*16*, *17*) (Fig. 1A). The N-terminal ExoN domain of nsp14 improves RNA synthesis fidelity by removing misincorporated nucleotides or nucleotide analogs from the nascent RNA, whereas the C-terminal N7-MTase domain is involved in the 5′ capping processes of the viral genomic and subgenomic mRNAs (*16*–*18*). The ExoN activity of nsp14 is stimulated by nsp10, which binds to the ExoN domain and helps stabilize the architecture of the ExoN active site (*18*). Previous studies of the SARS-CoV nsp10-nsp14 complex defined the nsp14 ExoN domain as a DED/EDh-type exonuclease and identified the five active-site residues through structural comparison and mutagenesis analyses (*19*). However, the molecular details of substrate binding by coronavirus nsp10-nsp14 ExoN remain unclear. In addition, how the viral ExoN recognizes and excises misincorporated nucleotides or nucleotide analog inhibitors at the 3′ end of the newly synthesized RNA is poorly understood.

To understand the substrate recognition and catalytic mechanism of SARS-CoV-2 ExoN, we constructed a hairpin RNA substrate (hereafter referred to as T35P31) that contains a template strand (T-strand, which is also the nonscissile strand for ExoN) with three initiating guanosines followed by the 32 nucleotides (nts) at the 3′ end of the SARS-CoV-2 genome [excluding the poly(A) tail] and a 31-nt product strand (P-strand, which is also the scissile strand for ExoN) ending with a cytidine-5′-monophosphate (CMP), resulting in a C-U mismatch at the 3′ end (Fig. 1C). The preformed SARS-CoV-2 nsp10-nsp14 complex digests the T35P31 RNA substrate in the presence of MgCl_2_ (Fig. 1B). To obtain a stable nsp10-nsp14-RNA complex, we substituted MgCl_2_ with CaCl_2_ in the reconstitution buffer or introduced an ExoN active-site mutation, E191A, to nsp14. Both measures retained the RNA binding capability but abolished the RNA cleavage activity of the nsp10-nsp14 complex (Fig. 1B and figs. S1 and S2).

The reconstituted nsp10-nsp14-RNA complexes were purified by size-exclusion chromatography (SEC) and analyzed by single-particle cryo–electron microscopy (cryo-EM). The final cryo-EM maps for the wild-type (WT) and mutant nsp10-nsp14-RNA complexes were refined to 3.9 Å (figs. S1 and S3) and 3.4 Å (figs. S2 and S3), respectively. With the exception of minor differences in the conformations of the RNA substrate and protein residue side chains, the two structures are almost identical, with a root mean square deviation of 0.39 Å across all protein Cα atoms (fig. S4). The ExoN active site, which is located in the nsp14 ExoN domain and supported by the N terminus of nsp10, binds the 3′ end of the RNA, separating it from the 5′ overhang (Fig. 1D). Most of the RNA helix remains freely accessible in the solvent-exposed space (Fig. 1D).

To explore the possible link between SARS-CoV-2 RdRp and ExoN, SARS-CoV-2 nsp8 was included in the reconstitution of the complex and was found to be coeluted with the nsp10-nsp14-RNA complex on the SEC column (figs. S1A and S2A). However, it did not form a stable complex with nsp10-nsp14-RNA in the cryo-EM sample and was observed in only a small fraction of the particles (fig. S2C), indicating that association of nsp8 with the nsp10-nsp14-RNA complex is weak and dynamic. Although further in silico classification of the nsp8-bound class did not yield a map with high-resolution features of nsp8, the 6-Å low-pass–filtered map showed strong extra density along the solvent exposed region of the RNA duplex (fig. S2C). When docking nsp8 from the SARS-CoV-2 RdRp complex structure (*20*) into the density as a rigid body, its N-terminal extended helices fit generally well, and its orientation relative to the RNA backbone matched that in the SARS-CoV-2 RdRp complex (*20*, *21*) (fig. S5A). The docking places the C-terminal domain of nsp8 outside of the cryo-EM density, but there is unoccupied cryo-EM density adjoining the N-terminal helices (fig. S5A), which suggests that nsp8 likely adopts a conformation that differs from when it is in the RdRp complex. This is consistent with previous structural studies, demonstrating extensive structural plasticity of nsp8 (*21*–*23*). The binding mode of nsp8 to the nsp10-nsp14-RNA complex suggests that nsp8 may help stabilize substrate binding for ExoN-mediated RNA cleavage. Indeed, an exoribonuclease activity assay shows that nsp8 enhances RNA digestion by the nsp10-nsp14 complex (fig. S5B). As a common component in both ExoN and RdRp complexes, nsp8 may play a role in RNA substrate transfer between the two enzymes. However, elucidating the detailed function of nsp8 in mismatch correction in vivo will require further investigation.

The cryo-EM sample reconstituted with the use of mutant ExoN contained a class that represents a tetrameric form of the nsp10-nsp14-RNA complex (Fig. 1E and fig. S2C). The tetramerization improved the resolution of three-dimensional (3D) reconstruction to 2.5 Å without affecting the architecture of the complex (figs. S2C and S6A). However, tetramerization of the nsp10-nsp14-RNA complex likely blocks nsp8 binding (fig. S6B). As a result, nsp8-like density is not observed along the RNA duplex in the tetramer map. Although 2D class averages from the WT nsp10-nsp14-RNA complex dataset also reveal particles that likely represent the tetrameric form of the complex (fig. S1B), the limited quantity of such particles precluded a meaningful 3D reconstruction. Unless otherwise indicated, we used the tetramer form of the nsp10-nsp14-RNA complexes for subsequent structural analyses of the ExoN active site and its interactions with RNA substrate because of its higher resolution.

**Fig. 2.**
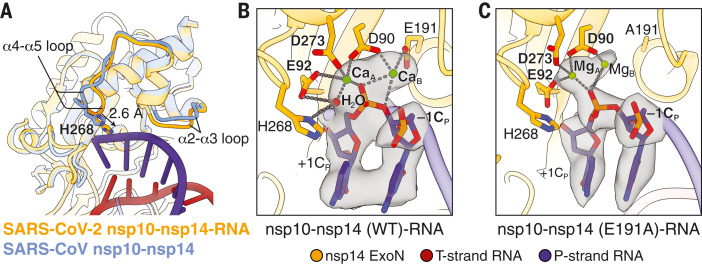
Active-site conformation and catalytic mechanism of SARS-CoV-2 ExoN. (**A**) Superimposition of the SARS-CoV nsp10-nsp14 complex (blue; PDB ID 5C8U) and the SARS-CoV-2 nsp10-nsp14-RNA complex (orange) illustrates the conformational changes of the α2-α3 and α4-α5 loops and a 2.6-Å shift of H268 toward the RNA upon substrate binding. (**B**) Active-site structure of the SARS-CoV-2 nsp10-nsp14 (WT)–RNA complex. Ca^2+^ ions, green spheres; catalytic water, red sphere. Nucleotide residues in P-strand RNA are indicated with a subscript “P.” +1C_P_, –1C_P_, the catalytic water, and two active-site metal ions are superimposed with their cryo-EM densities contoured at 10σ. (**C**) Active-site structure of the SARS-CoV-2 nsp10-nsp14 (E191A)–RNA complex. Mg^2+^ ions, green spheres. +1C_P_, –1C_P_, and two active-site metal ions are superimposed with their cryo-EM densities contoured at 7σ.

Compared with the apo form of the SARS-CoV nsp10-nsp14 complex (*19*), the structure of the SARS-CoV-2 WT nsp10-nsp14-RNA complex displays local conformational changes in the α4-α5 and α2-α3 loops, resulting in a slightly narrowed RNA binding pocket (Fig. 2A). Substrate binding also leads to full assembly of the ExoN active site. Whereas apo ExoN captures only one divalent metal ion (*19*), the RNA-bound ExoN contains two metal ion binding sites in its catalytic center (Fig. 2B and fig. S7A). Metal ion A, coordinated by carboxylate oxygens of D90, E92, and D273, activates a water molecule for nucleophilic attack. Metal ion B is coordinated by D90 and E191 and stabilizes the O3′ leaving group of –1C_P_ (nucleotide numbering shown in Fig. 1C) (Fig. 2B and fig. S7A). In the E191A mutant nsp10-nsp14-RNA complex, metal ion B is poorly coordinated, owing to the absence of the E191 side-chain carboxylate and is out of the coordination distance from the O3′ leaving group of –1C_P_ (Fig. 2C and fig. S7B). The fifth catalytic residue, H268, which functions as a general base and deprotonates the catalytic water during the phosphoryl-transfer reaction (*24*, *25*) (Fig. 2B and fig. S7A), is located in the nsp14 α4-α5 loop and shifts 2.6 Å toward the scissile phosphate, completing the active site in the presence of the RNA substrate (Fig. 2A).

The nucleoprotein (NP) of Lassa virus (LASV) in the Arenaviridae family represents the only other group of ExoNs found in RNA viruses (*14*, *15*, *26*). Although the coronavirus nsp14 and arenavirus NPs have evolved divergent additional domains to address different functions (*15*), the overall fold and active-site conformation of their ExoN domains are similar (fig. S7, C and D). The primary difference is that D466 of the LASV NP undertakes the role of E191 in nsp14 to coordinate metal ion B, presumably through an intermediate water molecule, owing to its shorter side chain (fig. S7D).

**Fig. 3.**
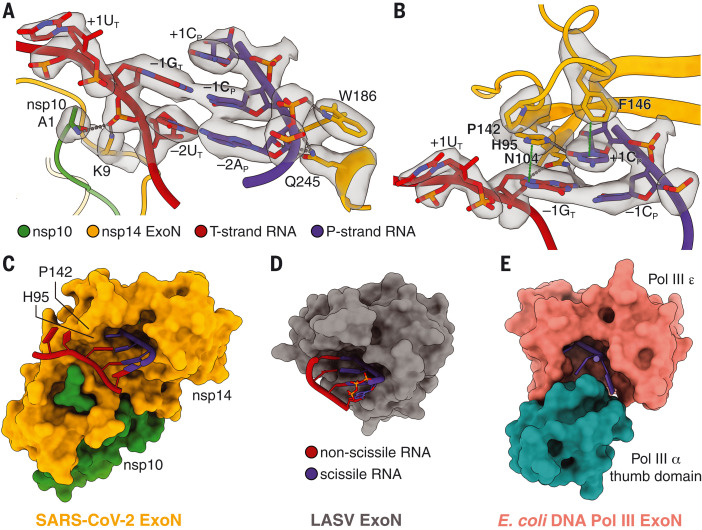
Mechanism of substrate recognition by SARS-CoV-2 ExoN. (**A**) Interactions between SARS-CoV-2 nsp10-nsp14 ExoN and the T35P31 RNA backbone. Nucleotide residues in T-strand RNA are indicated with a subscript “T,” whereas nucleotide residues in P-strand RNA are indicated with a subscript “P.” Hydrogen bonds and salt bridges are shown as gray dotted lines. Interacting nucleotide and protein residues are superimposed with their cryo-EM densities contoured at 7σ. (**B**) Interactions between SARS-CoV-2 nsp10-nsp14 ExoN and T35P31 RNA at +1 and –1 nucleobase positions. Hydrogen bonds and salt bridges are shown as gray dotted lines. π-π stacking interactions are indicated by green dotted lines. Interacting nucleotide and protein residues are superimposed with their cryo-EM densities contoured at 7σ. (**C**) Surface representation of the SARS-CoV-2 nsp10-nsp14 ExoN substrate binding pocket shows a restricted opening on the T-strand side that prevents base-pairing at the substrate RNA +1 position. For clarity, the N7-MTase domain of nsp14 is not shown. (**D**) Surface representation of the LASV NP ExoN domain. A fully base-paired dsRNA substrate (shown in cartoon representation) is bound in the substrate binding pocket of LASV ExoN. (**E**) Surface representation of the *E. coli* DNA Pol III ExoN complex. The narrow substrate binding pocket allows the entry of ssDNA only. F, Phe; K, Lys; N, Asn; P, Pro; Q, Gln; W, Trp.

The shallow SARS-CoV-2 ExoN substrate binding pocket encompasses only base pairs –1 and –2 of the dsRNA, interacting with the RNA backbone through the A1 of nsp10 and K9, W186, and Q245 in nsp14 (Fig. 3A). At the 3′ end of the dsRNA substrate, nsp14 separates the mismatched C-U pair and flips +1U_T_ out of the RNA double helix (Fig. 3, A and B). As a result, binding in the SARS-CoV-2 ExoN active site is a dsRNA with a 1-nt 3′ overhang comprising +1C_P_ (Fig. 3, A and B), a substrate structure different from that observed in other RNA virus and proofreading DED/EDh exonucleases (*26*–*28*) and from that previously predicted for SARS-CoV ExoN (*8*, *18*). The substrate specificity of SARS-CoV-2 ExoN is contributed by many interactions between nsp14 and the RNA substrate (Fig. 3, A and B). F146 at the bottom of the SARS-CoV-2 ExoN substrate binding pocket stacks against the 3′-end unpaired +1C_P_. N104 inserts into the minor groove of the dsRNA and establishes two hydrogen bonds with the nucleobase and 2′-OH group of –1G_T_, respectively. H95, which is approximately coplanar with the unpaired +1C_P_, is hydrogen-bonded with the cytidine base and stacks against –1G_T_ (Fig. 3B). The ability of H95 to act as both hydrogen bond donor and acceptor probably allows it to accommodate all four types of nucleotides, explaining the relative insensitivity of nsp14 to substrate sequence (*18*). Digestion of dsRNA substrates by SARS-CoV-2 ExoN may slow at a C-G base pair, owing to the higher energy required to break this base pair. Additionally, P142, situated at the rim of the ExoN RNA binding pocket, works together with H95 to restrict the depth of the substrate binding pocket on the T-strand side and likely forces the strand separation of the RNA substrate 3′-end C-U mismatched pair (Fig. 3, B and C). The lower energy needed for separating a mismatched base pair could explain the preference of coronavirus ExoN for a dsRNA substrate with a 3′-end mismatch instead of a perfectly matched substrate (*18*). By contrast, the LASV ExoN RNA binding pocket has a slightly deeper opening on the nonscissile strand side and therefore is able to accommodate a fully base-paired dsRNA substrate (*26*) (Fig. 3D). This is consistent with its role as a dsRNA-degrading immune suppressor rather than an RNA synthesis proofreader (*14*, *15*). At the other end of the spectrum are DNA polymerase–associated proofreading ExoNs, such as the *Escherichia coli* DNA polymerase III (Pol III) ε subunit. This subunit has a much narrower DNA binding pocket, partially because of its tight association with the Pol III α subunit, and can fit only a single-stranded DNA substrate (*27*) (Fig. 3E). All of the RNA-contacting residues in nsp14 are highly conserved among different coronavirus genera (fig. S8), indicating a shared RNA substrate recognition mechanism of coronavirus ExoN.

**Fig. 4.**
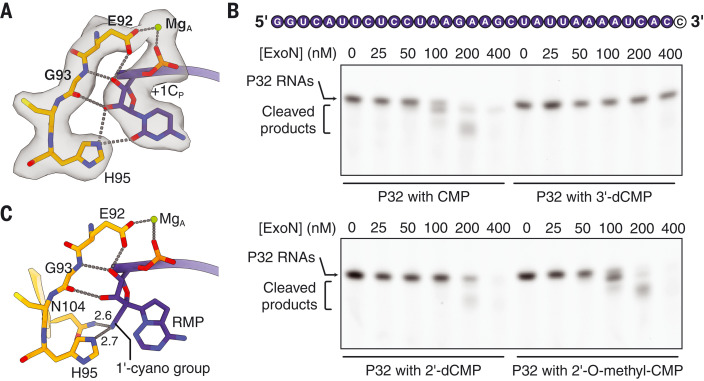
Structural insights into antiviral design. (**A**) Interactions between SARS-CoV-2 nsp10-nsp14 ExoN and the 3′-end nucleotide mediated by its 2′- and 3′-OH groups. Mg^2+^ ion, green sphere. Hydrogen bonds are shown as gray dotted lines. +1C_P_ and its interacting protein residues are superimposed with cryo-EM densities contoured at 7σ. (**B**) Cleavage of various P32 ssRNA substrates by the SARS-CoV-2 nsp10-nsp14 complex. The 3′-end CMP that bears different modifications at its 2′- or 3′-OH groups is shown as a black letter in a white circle. The nanomolar concentrations of ExoN are indicated. The RNAs were resolved by denaturing PAGE and stained by SYBR Gold. A representative result from three biological replicates is shown. (**C**) Predicted interactions between SARS-CoV-2 nsp10-nsp14 ExoN and RMP, which is modeled at the 3′-end +1 position of P-strand RNA. G, Gly.

As a 3′-to-5′ exoribonuclease, SARS-CoV-2 nsp10-nsp14 specifically recognizes the 2′- and 3′-OH groups of the 3′-end nucleotide. The 2′-OH of +1C_P_ forms two hydrogen bonds with H95 and the carbonyl oxygen of G93, respectively, whereas the 3′-OH of the nucleotide is hydrogen bonded with the G93 main-chain nitrogen and catalytic residue E92 (Fig. 4A). To examine the effects of the 2′- and 3′-OH groups of the 3′-end nucleotide on RNA cleavage efficiency by SARS-CoV-2 ExoN, we performed exonuclease assays using 32-nt single-stranded RNA (ssRNA) substrates (referred to as P32 RNAs) ending with either a standard ribonucleotide or a nucleotide with modifications at the 2′ or 3′ position (Fig. 4B). SARS-CoV-2 nsp10-nsp14 efficiently cleaves the unmodified ssRNA, although significantly higher enzyme concentrations are needed to obtain cleavage comparable to that achieved on a dsRNA substrate with the same P-strand sequence (fig. S9). This is likely due to the weaker binding of ssRNA to SARS-CoV-2 ExoN, resulting from the loss of protein-RNA interactions on the T-strand side (Fig. 3, A and B). The ability of ExoN to accept both ssRNA and dsRNA substrates suggests two possible modes of mismatch correction in vivo. ExoN may bind to and cleave the 3′-end single-stranded region of P-strand RNA, which resulted from RdRp backtracking, as proposed by previous studies (*21*, *29*). Alternatively, dsRNA substrates that contain a 3′-end mismatch may dissociate from RdRp and are subsequently recognized by ExoN for mismatch excision.

Removing the 2′- or 3′-OH groups of the 3′-end nucleotide either reduces or almost abolishes nucleolytic degradation by SARS-CoV-2 ExoN within the range of tested enzyme concentrations (Fig. 4B), consistent with the previous findings on SARS-CoV ExoN (*18*) and reflecting the key roles of 2′- and 3′-oxygens in coronavirus ExoN catalysis. On the other hand, 2′-O-methylation of the 3′-end cytidine does not substantially affect the substrate cleavage by SARS-CoV-2 nsp10-nsp14 (Fig. 4B), likely because some interactions between the 2′-oxygen and nsp14 are retained.

Remdesivir is the only US Food and Drug Administration–approved nucleotide analog antiviral to treat COVID-19. To assess whether remdesivir can be effectively excised by SARS-CoV-2 ExoN, we modeled the incorporated form of the inhibitor, remdesivir monophosphate (RMP), at the +1 position of the P-strand (Fig. 4C). The modeled RMP maintains most of the favorable interactions formed between nsp14 and the 3′-end CMP. In addition, the 1′-cyano group of RMP, the determinant of its delayed RdRp stalling activity (*6*, *7*), snugly fits in the space between H95 and N104 and forms hydrogen bonds with the side-chain nitrogen atoms from the two residues (Fig. 4C). These observations indicate that product RNA containing RMP could be a substrate for coronavirus ExoN, consistent with the findings that RNA terminated with RMP does not display substantial resistance to ExoN excision (*30*) and that coronaviruses lacking ExoN proofreading activity are significantly more sensitive to remdesivir (*31*).

Our study offers insights into the mechanism of mismatch correction during SARS-CoV-2 RNA synthesis and reveals the structural features in the substrate that are essential for ExoN recognition and catalysis, providing a basis for structural-guided design of specific and potent ExoN inhibitors. Coadministration of such ExoN inhibitors with nucleotide analog–based viral RdRp antivirals could constitute a more effective treatment for COVID-19. Additionally, our study sheds light on the development of ExoN-resistant nucleotide analog inhibitors. In particular, we show that a free 3′-OH of the RNA substrate is critical for exonucleolytic degradation by ExoN. It has been shown that 3′-deoxy ribonucleotides can be efficiently incorporated into nascent RNA by RdRp from other positive-strand RNA viruses, such as hepatitis C virus and poliovirus, and can subsequently block RNA extension (*32*, *33*). Therefore, 3′-deoxy nucleotide analogs can potentially act as effective coronavirus RdRp chain terminators that also resist ExoN excision. Nonetheless, modifications at other positions on the ribose ring also merit further exploration.

## Supplementary Material

20210727-1

## Supplementary Materials

science.sciencemag.org/content/373/6559/1142/suppl/DC1
Materials and Methods Figs. S1 to S9 Table S1 References (*34*–*47*) MDAR Reproducibility Checklist

## Appendix

View/request a protocol for this paper from *Bio-protocol*.
