# Supplementary material for: Structural basis of mismatch recognition by a SARS-CoV-2 proofreading enzyme
Source: Science. 2021 Jul 27;373(6559):1142–6. doi: 10.1126/science.abi9310 (PMC9836006; doi:10.1126/science.abi9310)
Supplement: 20210727-1 [file science.abi9310.v1.pdf]

Cite as: C. Liu *et al.*, *Science*
10.1126/science.abi9310 (2021).

Structural basis of mismatch recognition by a SARS-CoV-2 proofreading enzyme

Chang Liu[1]*, Wei Shi[2], Scott T. Becker[3], David G. Schatz[1], Bin Liu[2]*, Yang Yang[3]*

[1]Department of Immunobiology, Yale School of Medicine, New Haven, CT, USA. [2]Section of Transcription & Gene Regulation, The Hormel Institute, University of Minnesota, Austin, MN, USA. [3]Roy J. Carver Department of Biochemistry, Biophysics and Molecular Biology, Iowa State University, Ames, IA, USA.

***Corresponding author. Email: c.liu@yale.edu (C.L.); liu00794@umn.edu (B.L.); yan9yang@iastate.edu (Y.Y.)**

Coronavirus 3′–5′ exoribonuclease (ExoN), residing in the nonstructural protein (nsp) 10-nsp14 complex, boosts replication fidelity by proofreading RNA synthesis and is critical for the virus life cycle. ExoN also recognizes and excises nucleotide analog inhibitors incorporated into the nascent RNA, undermining the effectiveness of nucleotide analog-based antivirals. Here, we present cryo-electron microscopy structures of both wild-type and mutant SARS-CoV-2 nsp10-nsp14 in complex with an RNA substrate bearing a 3′-end mismatch at resolutions ranging from 2.5 Å to 3.9 Å. The structures reveal the molecular determinants of ExoN substrate specificity and give insight into the molecular mechanisms of mismatch correction during coronavirus RNA synthesis. Our findings provide guidance for rational design of improved anti-coronavirus therapies.

SARS-CoV-2, the causative agent of the COVID-19 pandemic, has infected over 160 million people and led to over 3 million deaths worldwide (https://covid19.who.int). Although several SARS-CoV-2 vaccines are now available (*1*), there are no highly effective antiviral agents to treat the disease. One of the most important druggable targets for SARS-CoV-2 is its replication/transcription complex (RTC), a multi-subunit machine that carries out viral genome replication and transcription and plays an essential role in the virus life cycle (*2*, *3*). Central to the coronavirus RTC is the core RNA-dependent RNA polymerase (RdRp), nsp12 (*4*), and two associated accessory proteins, nsp7 and nsp8 (*5*). SARS-CoV-2 RdRp is a promising target for nucleotide analog antivirals, such as remdesivir (*6*, *7*). However, the efficacy of nucleotide analog inhibitors on coronavirus RdRp is compromised by the presence of the viral nsp14 exoribonuclease (ExoN) (*8*, *9*), an RNA proofreader specific to coronaviruses and a few other closely related virus families of the Nidovirales order and crucial to maintain the integrity of their unusually large RNA genome (*9*–*11*). In addition, ExoNs from coronaviruses and other RNA viruses play an important role in the evasion of host immune responses by degrading the viral double-stranded RNA (dsRNA) intermediates that would otherwise be recognized by host pathogen recognition receptors (*12*–*15*).

Nsp14 is a bi-functional enzyme that harbors both 3′-5′ ExoN and mRNA cap guanine-N7 methyltransferase (N7-MTase) activities (*16*, *17*) (Fig. 1A). The N-terminal ExoN domain of nsp14 improves RNA synthesis fidelity by removing mis-incorporated nucleotides or nucleotide analogs from the nascent RNA, while the C-terminal N7-MTase domain is involved in the 5′ capping processes of the viral genomic and subgenomic messenger RNAs (*16*–*18*). The ExoN activity of nsp14 is stimulated by nsp10, which binds to the ExoN domain and helps stabilize the architecture of the ExoN active site (*18*). Previous studies of the SARS-CoV nsp10-nsp14 complex defined the nsp14 ExoN domain as a DED/EDh-type exonuclease and identified the five active site residues through structural comparison and mutagenesis analyses (*19*). However, the molecular details of substrate binding by coronavirus nsp10-nsp14 ExoN remain unclear. In addition, how the viral ExoN recognizes and excises mis-incorporated nucleotide or nucleotide analog inhibitor at the 3′ end of the newly synthesized RNA is poorly understood.

To understand the substrate recognition and catalytic mechanism of SARS-CoV-2 ExoN, we constructed a hairpin RNA substrate (hereafter referred to as T35P31) that contains a template strand (T-strand, which is also the non-scissile strand for ExoN) with three initiating guanosines followed by the 3′-end 32 nucleotides (nt) of the SARS-CoV-2 genome (excluding the poly(A) tail) and a 31-nt product strand (P-strand, which is also the scissile strand for ExoN) ending with a cytidine-5′-monophosphate (CMP), resulting in a C-U mismatch at the 3′ end (Fig. 1C). The pre-formed SARS-CoV-2 nsp10-nsp14 complex digests the T35P31 RNA substrate in the presence of $MgCl_2$ (Fig. 1B). To obtain a stable nsp10-nsp14-RNA complex, we substituted $MgCl_2$ with $CaCl_2$ in the reconstitution buffer, or introduced an ExoN active site mutation, E191A, to nsp14. Both measures retained the RNA-binding capability but abolished the RNA cleavage activity of the nsp10-nsp14 complex (Fig. 1B and figs. S1 and S2).

The reconstituted nsp10-nsp14-RNA complexes were purified by size-exclusion chromatography (SEC) and analyzed by

single-particle cryo-EM. The final cryo-EM maps for the WT and mutant nsp10-nsp14-RNA complexes were refined to 3.9 Å (figs. S1 and S3) and 3.4 Å (figs. S2 and S3), respectively. With the exception of minor differences in the conformations of the RNA substrate and protein residue side chains, the two structures are almost identical with an RMSD of 0.39 Å across all protein Cα atoms (fig. S4). The ExoN active site, which is located in nsp14 ExoN domain and supported by the N terminus of nsp10, binds the 3′ end of the RNA, separating it from the 5′ overhang (Fig. 1D). The majority of the RNA helix remains freely accessible in the solvent-exposed space (Fig. 1D).

To explore the possible link between SARS-CoV-2 RdRp and ExoN, SARS-CoV-2 nsp8 was included in the reconstitution of the complex and was found to be co-eluted with the nsp10-nsp14-RNA complex on the SEC column (figs. S1A and S2A). However, it did not form a stable complex with nsp10-nsp14-RNA in the cryo-EM sample and was only observed in a small fraction of the particles (fig. S2C), indicating that association of nsp8 with the nsp10-nsp14-RNA complex is weak and dynamic. Although further *in silico* classification of the nsp8-bound class did not yield a map with high-resolution features of nsp8, the 6 Å low-pass filtered map showed strong extra density along the solvent exposed region of the RNA duplex (fig. S2C). When docking nsp8 from the SARS-CoV-2 RdRp complex structure (*20*) into the density as a rigid body, its N-terminal extended helices fit generally well and its orientation relative to the RNA backbone matched that in the SARS-CoV-2 RdRp complex (*20*, *21*) (fig. S5A). The docking places the C-terminal domain of nsp8 outside of the cryo-EM density, but there is unoccupied cryo-EM density adjoining the N-terminal helices (fig. S5A), suggesting nsp8 likely adopts a different conformation than when it is in the RdRp complex. This is consistent with previous structural studies, demonstrating extensive structural plasticity of nsp8 (*21*–*23*). The binding mode of nsp8 to nsp10-nsp14-RNA complex suggests nsp8 may help stabilize substrate binding for ExoN-mediated RNA cleavage. Indeed, exoribonuclease activity assay shows nsp8 enhances RNA digestion by the nsp10-nsp14 complex (fig. S5B). As a common component in both ExoN and RdRp complexes, nsp8 may play a role in RNA substrate transfer between the two enzymes. However, the detailed function of nsp8 in mismatch correction in vivo needs further investigation.

The cryo-EM sample reconstituted using mutant ExoN contained a class that represents a tetrameric form of the nsp10-nsp14-RNA complex (Fig. 1E and fig. S2C). The tetramerization improved the resolution of 3D reconstruction to 2.5 Å without affecting the architecture of the complex (figs. S2C and S6A). However, tetramerization of nsp10-nsp14-RNA complex likely blocks nsp8 binding (fig. S6B). As a result, nsp8-like density is not observed along the RNA duplex in the tetramer map. Although 2D class averages from the WT nsp10-nsp14-RNA complex dataset also reveal particles likely representing the tetrameric form of the complex (fig. S1B), the limited quantity of such particles precluded a meaningful 3D reconstruction. Unless otherwise indicated, we will use the tetramer form of the nsp10-nsp14-RNA complexes for subsequent structural analyses of the ExoN active site and its interactions with RNA substrate because of its higher resolution.

Compared with the apo form of the SARS-CoV nsp10-nsp14 complex (*19*), the structure of SARS-CoV-2 WT nsp10-nsp14-RNA complex displays local conformational changes in the α4-α5 and α2-α3 loops, resulting in a slightly narrowed RNA-binding pocket (Fig. 2A). Substrate binding also leads to full assembly of the ExoN active site. While apo ExoN captures only one divalent metal ion (*19*), the RNA-bound ExoN contains two metal ion binding sites in its catalytic center (Fig. 2B and fig. S7A). Metal ion A, coordinated by carboxylate oxygens of D90, E92 and D273, activates a water molecule for nucleophilic attack. Metal ion B is coordinated by D90 and E191 and stabilizes the O3′ leaving group of $-1C_P$ (nucleotide numbering shown in Fig. 1C) (Fig. 2B and fig. S7A). In the E191A mutant nsp10-nsp14-RNA complex, metal ion B is poorly coordinated due to the absence of E191 side chain carboxylate and is out of the coordination distance from the O3′ leaving group of $-1C_P$ (Fig. 2C and fig. S7B). The fifth catalytic residue H268, which functions as a general base and deprotonates the catalytic water during the phosphoryl transfer reaction (*24*, *25*) (Fig. 2B and fig. S7A), is located in the nsp14 α4-α5 loop and shifts 2.6 Å toward the scissile phosphate, completing the active site in the presence of the RNA substrate (Fig. 2A).

The nucleoprotein (NP) of Lassa virus (LASV) in the Arenaviridae family represents the only other group of ExoNs found in RNA viruses (*14*, *15*, *26*). Although the coronavirus nsp14 and arenaviruses NP have evolved divergent additional domains to address different functions (*15*), the overall fold and active site conformation of their ExoN domains are similar (fig. S7, C and D). The major difference is that D466 of LASV NP undertakes the role of E191 in nsp14 to coordinate metal ion B, presumably through an intermediate water molecule due to its shorter side chain (fig. S7D).

The shallow SARS-CoV-2 ExoN substrate-binding pocket encompasses only base pairs (bp) −1 and −2 of the dsRNA, interacting with the RNA backbone through the A1 of nsp10 and K9, W186 and Q245 in nsp14 (Fig. 3A). At the 3′ end of the dsRNA substrate, nsp14 separates the mismatched C-U pair and flips $+1U_T$ out of the RNA double helix (Fig. 3, A and B). As a result, binding in the SARS-CoV-2 ExoN active site is a dsRNA with 1-nt 3′ overhang comprising $+1C_P$ (Fig. 3, A and B), a substrate structure different from that observed in other RNA virus and proofreading DED/EDh exonucleases (*26*–*28*) and from previously predicted for SARS-CoV ExoN (*8*, *18*).

The substrate specificity of SARS-CoV-2 ExoN is contributed by many interactions between nsp14 and the RNA substrate (Fig. 3, A and B). F146 at the bottom of the SARS-CoV-2 ExoN substrate-binding pocket stacks against the 3′-end unpaired $+1C_P$. N104 inserts into the minor groove of the dsRNA and establishes two hydrogen bonds with the nucleobase and 2′-OH group of $-1G_T$, respectively. H95, which is approximately co-planar with the unpaired $+1C_P$, is hydrogen bonded with the cytidine base and stacks against $-1G_T$ (Fig. 3B). The ability of H95 to act as both hydrogen bond donor and acceptor probably allows it to accommodate all four types of nucleotides, explaining the relative insensitivity of nsp14 to substrate sequence (*18*). Digestion of dsRNA substrates by SARS-CoV-2 ExoN may slow at a C-G base pair due to the higher energy required to break this base pair. Additionally, P142, situated at the rim of the ExoN RNA-binding pocket, works together with H95 to restrict the depth of the substrate-binding pocket on the T-strand side and likely forces the strand separation of the RNA substrate 3′-end C-U mismatched pair (Fig. 3, B and C). The lower energy for separating a mismatched base pair could explain the preference of coronavirus ExoN for dsRNA substrate with a 3′-end mismatch over a perfectly matched substrate (*18*). By contrast, the LASV ExoN RNA-binding pocket has a slightly deeper opening on the non-scissile strand side and therefore is able to accommodate a fully base-paired dsRNA substrate (*26*) (Fig. 3D). This is consistent with its role as an dsRNA-degrading immune suppressor, rather than an RNA synthesis proofreader (*14*, *15*). At the other end of the spectrum, are DNA polymerase-associated proofreading ExoNs, such as the *E. coli* DNA polymerase III (Pol III) ε subunit. It has a much narrower DNA-binding pocket, partially due to its tight association with the Pol III α subunit, and can only fit a single-stranded DNA substrate (*27*) (Fig. 3E). All the RNA-contacting residues in nsp14 are highly conserved among different coronavirus genera (fig. S8), indicating a shared RNA substrate recognition mechanism of coronavirus ExoN.

As a 3′–5′ exoribonuclease, SARS-CoV-2 nsp10-nsp14 specifically recognizes the 2′- and 3′-OH groups of the 3′-end nucleotide. The 2′-OH of $+1C_P$ forms two hydrogen bonds with H95 and the carbonyl oxygen of G93, respectively, whereas the 3′-OH of the nucleotide is hydrogen bonded with the G93 main chain nitrogen and catalytic residue E92 (Fig. 4A). To examine the effects of the 2′- and 3′-OH groups of the 3′-end nucleotide on RNA cleavage efficiency by SARS-CoV-2 ExoN, we performed the exonuclease assays using 32-nt single-stranded RNA (ssRNA) substrates (referred to as P32 RNAs) ending with either a standard ribonucleotide or a nucleotide with modifications at the 2′ or 3′ position (Fig. 4B). SARS-CoV-2 nsp10-nsp14 efficiently cleaves the unmodified ssRNA, although significantly higher enzyme concentrations are needed to obtain cleavage comparable to that achieved on a dsRNA substrate with the same P-strand sequence (fig. S9). This is likely due to the weaker binding of ssRNA to SARS-CoV-2 ExoN resulting from the loss of protein-RNA interactions on the T-strand side (Fig. 3, A and B). The ability of ExoN to accept both ssRNA and dsRNA substrates suggest two possible modes of mismatch correction in vivo. ExoN may bind to and cleave the 3′-end single-stranded region of P-strand RNA resulted from RdRp backtracking, as proposed by previous studies (*21*, *29*). Alternatively, the dsRNA substrates containing a 3′-end mismatch may dissociate from RdRp and are subsequently recognized by ExoN for mismatch excision.

Removing the 2′- or 3′-OH groups of the 3′-end nucleotide either reduces or almost abolishes nucleolytic degradation by SARS-CoV-2 ExoN within the range of tested enzyme concentrations (Fig. 4B), consistent with the previous findings on SARS-CoV ExoN (*18*) and reflecting the important roles of 2′- and 3′-oxygens in coronavirus ExoN catalysis. On the other hand, 2′-O-methylation of the 3′-end cytidine does not significantly affect the substrate cleavage by SARS-CoV-2 nsp10-nsp14 (Fig. 4B), likely because some interactions between the 2′-oxygen and nsp14 are retained.

Remdesivir is the only FDA-approved nucleotide analog antiviral to treat COVID-19. To assess if remdesivir can be effectively excised by SARS-CoV-2 ExoN, we modeled the incorporated form of the inhibitor, remdesivir monophosphate (RMP), at the +1 position of the P-strand (Fig. 4C). The modeled RMP maintains most of the favorable interactions formed between nsp14 and the 3′-end CMP. In addition, the 1′-cyano group of RMP, the determinant of its delayed RdRp stalling activity (*6*, *7*), snugly fits in the space between H95 and N104 and forms hydrogen bonds with the side chain nitrogen atoms from the two residues (Fig. 4C). These observations indicate that product RNA containing RMP could be a substrate for coronavirus ExoN, consistent with the findings that RNA terminated with RMP does not display significant resistance to ExoN excision (*30*) and that coronaviruses lacking ExoN proofreading activity was significantly more sensitive to remdesivir (*31*).

Our study gives insights into the mechanism of mismatch correction during SARS-CoV-2 RNA synthesis and reveals the structural features in the substrate that are essential for ExoN recognition and catalysis, providing a basis for structural-guided design of specific and potent ExoN inhibitors. Coadministration of such ExoN inhibitors with nucleotide analog-based viral RdRp antivirals could constitute a more effective treatment for COVID-19. Additionally, our study sheds light on the development of ExoN-resistant nucleotide analog inhibitors. In particular, we show that a free 3′-OH of the RNA substrate is critical for exonucleolytic degradation by ExoN. It has been shown that 3′-deoxy ribonucleotides can be efficiently incorporated into nascent RNA by RdRp from other

positive-strand RNA viruses, such as HCV and poliovirus, and subsequently block RNA extension (*32*, *33*). Therefore, 3′-deoxy nucleotide analogs can potentially act as effective coronavirus RdRp chain terminators that also resist ExoN excision. Nonetheless, modifications at other positions on the ribose ring are also worth further exploration.

REFERENCES AND NOTES

1. A. Mullard, COVID-19 vaccines buoy hope. *Nat. Rev. Drug Discov.* **20**, 8 (2021). doi:10.1038/d41573-020-00215-9 Medline
2. J. Ziebuhr, The coronavirus replicase. *Curr. Top. Microbiol. Immunol.* **287**, 57–94 (2005). doi:10.1007/3-540-26765-4_3 Medline
3. I. Sola, F. Almazán, S. Zúñiga, L. Enjuanes, Continuous and Discontinuous RNA Synthesis in Coronaviruses. *Annu. Rev. Virol.* **2**, 265–288 (2015). doi:10.1146/annurev-virology-100114-055218 Medline
4. A. J. te Velthuis, J. J. Arnold, C. E. Cameron, S. H. van den Worm, E. J. Snijder, The RNA polymerase activity of SARS-coronavirus nsp12 is primer dependent. *Nucleic Acids Res.* **38**, 203–214 (2010). doi:10.1093/nar/gkp904 Medline
5. Y. Gao, L. Yan, Y. Huang, F. Liu, Y. Zhao, L. Cao, T. Wang, Q. Sun, Z. Ming, L. Zhang, J. Ge, L. Zheng, Y. Zhang, H. Wang, Y. Zhu, C. Zhu, T. Hu, T. Hua, B. Zhang, X. Yang, J. Li, H. Yang, Z. Liu, W. Xu, L. W. Guddat, Q. Wang, Z. Lou, Z. Rao, Structure of the RNA-dependent RNA polymerase from COVID-19 virus. *Science* **368**, 779–782 (2020). doi:10.1126/science.abb7498 Medline
6. G. Kokic, H. S. Hillen, D. Tegunov, C. Dienemann, F. Seitz, J. Schmitzova, L. Farnung, A. Siewert, C. Höbartner, P. Cramer, Mechanism of SARS-CoV-2 polymerase stalling by remdesivir. *Nat. Commun.* **12**, 279 (2021). doi:10.1038/s41467-020-20542-0 Medline
7. J. P. K. Bravo, T. L. Dangerfield, D. W. Taylor, K. A. Johnson, Remdesivir is a delayed translocation inhibitor of SARS-CoV-2 replication. *Mol. Cell* **81**, 1548–1552.e4 (2021). doi:10.1016/j.molcel.2021.01.035 Medline
8. F. Ferron, L. Subissi, A. T. Silveira De Morais, N. T. T. Le, M. Sevajol, L. Gluais, E. Decroly, C. Vonrhein, G. Bricogne, B. Canard, I. Imbert, Structural and molecular basis of mismatch correction and ribavirin excision from coronavirus RNA. *Proc. Natl. Acad. Sci. U.S.A.* **115**, E162–E171 (2018). doi:10.1073/pnas.1718806115 Medline
9. F. Robson, K. S. Khan, T. K. Le, C. Paris, S. Demirbag, P. Barfuss, P. Rocchi, W.-L. Ng, Coronavirus RNA Proofreading: Molecular Basis and Therapeutic Targeting. *Mol. Cell* **79**, 710–727 (2020). doi:10.1016/j.molcel.2020.07.027 Medline
10. A. E. Gorbalenya, L. Enjuanes, J. Ziebuhr, E. J. Snijder, Nidovirales: Evolving the largest RNA virus genome. *Virus Res.* **117**, 17–37 (2006). doi:10.1016/j.virusres.2006.01.017 Medline
11. N. S. Ogando, J. C. Zevenhoven-Dobbe, Y. van der Meer, P. J. Bredenbeek, C. C. Posthuma, E. J. Snijder, The Enzymatic Activity of the nsp14 Exoribonuclease Is Critical for Replication of MERS-CoV and SARS-CoV-2. *J. Virol.* **94**, e01246-20 (2020). doi:10.1128/JVI.01246-20 Medline
12. M. Becares, A. Pascual-Iglesias, A. Nogales, I. Sola, L. Enjuanes, S. Zuñiga, Mutagenesis of Coronavirus nsp14 Reveals Its Potential Role in Modulation of the Innate Immune Response. *J. Virol.* **90**, 5399–5414 (2016). doi:10.1128/JVI.03259-15 Medline
13. J. B. Case, Y. Li, R. Elliott, X. Lu, K. W. Graepel, N. R. Sexton, E. C. Smith, S. R. Weiss, M. R. Denison, Murine Hepatitis Virus nsp14 Exoribonuclease Activity Is Required for Resistance to Innate Immunity. *J. Virol.* **92**, e01531-17 (2017). doi:10.1128/JVI.01531-17 Medline
14. K. M. Hastie, C. R. Kimberlin, M. A. Zandonatti, I. J. MacRae, E. O. Saphire, Structure of the Lassa virus nucleoprotein reveals a dsRNA-specific 3′ to 5′ exonuclease activity essential for immune suppression. *Proc. Natl. Acad. Sci. U.S.A.* **108**, 2396–2401 (2011). doi:10.1073/pnas.1016404108 Medline
15. X. Qi, S. Lan, W. Wang, L. M. L. Schelde, H. Dong, G. D. Wallat, H. Ly, Y. Liang, C. Dong, Cap binding and immune evasion revealed by Lassa nucleoprotein structure. *Nature* **468**, 779–783 (2010). doi:10.1038/nature09605 Medline
16. E. Minskaia, T. Hertzig, A. E. Gorbalenya, V. Campanacci, C. Cambillau, B. Canard, J. Ziebuhr, Discovery of an RNA virus 3′->5′ exoribonuclease that is critically involved in coronavirus RNA synthesis. *Proc. Natl. Acad. Sci. U.S.A.* **103**, 5108–5113 (2006). doi:10.1073/pnas.0508200103 Medline
17. Y. Chen, H. Cai, J. Pan, N. Xiang, P. Tien, T. Ahola, D. Guo, Functional screen reveals SARS coronavirus nonstructural protein nsp14 as a novel cap N7 methyltransferase. *Proc. Natl. Acad. Sci. U.S.A.* **106**, 3484–3489 (2009). doi:10.1073/pnas.0808790106 Medline
18. M. Bouvet, I. Imbert, L. Subissi, L. Gluais, B. Canard, E. Decroly, RNA 3′-end mismatch excision by the severe acute respiratory syndrome coronavirus nonstructural protein nsp10/nsp14 exoribonuclease complex. *Proc. Natl. Acad. Sci. U.S.A.* **109**, 9372–9377 (2012). doi:10.1073/pnas.1201130109 Medline
19. Y. Ma, L. Wu, N. Shaw, Y. Gao, J. Wang, Y. Sun, Z. Lou, L. Yan, R. Zhang, Z. Rao, Structural basis and functional analysis of the SARS coronavirus nsp14-nsp10 complex. *Proc. Natl. Acad. Sci. U.S.A.* **112**, 9436–9441 (2015). doi:10.1073/pnas.1508686112 Medline
20. H. S. Hillen, G. Kokic, L. Farnung, C. Dienemann, D. Tegunov, P. Cramer, Structure of replicating SARS-CoV-2 polymerase. *Nature* **584**, 154–156 (2020). doi:10.1038/s41586-020-2368-8 Medline
21. J. Chen, B. Malone, E. Llewellyn, M. Grasso, P. M. M. Shelton, P. D. B. Olinares, K. Maruthi, E. T. Eng, H. Vatandaslar, B. T. Chait, T. M. Kapoor, S. A. Darst, E. A. Campbell, Structural Basis for Helicase-Polymerase Coupling in the SARS-CoV-2 Replication-Transcription Complex. *Cell* **182**, 1560–1573.e13 (2020). doi:10.1016/j.cell.2020.07.033 Medline
22. Y. Zhai, F. Sun, X. Li, H. Pang, X. Xu, M. Bartlam, Z. Rao, Insights into SARS-CoV transcription and replication from the structure of the nsp7-nsp8 hexadecamer. *Nat. Struct. Mol. Biol.* **12**, 980–986 (2005). doi:10.1038/nsmb999 Medline
23. Y. Xiao, Q. Ma, T. Restle, W. Shang, D. I. Svergun, R. Ponnusamy, G. Sczakiel, R. Hilgenfeld, Nonstructural proteins 7 and 8 of feline coronavirus form a 2:1 heterotrimer that exhibits primer-independent RNA polymerase activity. *J. Virol.* **86**, 4444–4454 (2012). doi:10.1128/JVI.06635-11 Medline
24. Y. Zuo, M. P. Deutscher, Exoribonuclease superfamilies: Structural analysis and phylogenetic distribution. *Nucleic Acids Res.* **29**, 1017–1026 (2001). doi:10.1093/nar/29.5.1017 Medline
25. S. Hamdan, P. D. Carr, S. E. Brown, D. L. Ollis, N. E. Dixon, Structural basis for proofreading during replication of the Escherichia coli chromosome. *Structure* **10**, 535–546 (2002). doi:10.1016/S0969-2126(02)00738-4 Medline
26. X. Jiang, Q. Huang, W. Wang, H. Dong, H. Ly, Y. Liang, C. Dong, Structures of arenaviral nucleoproteins with triphosphate dsRNA reveal a unique mechanism of immune suppression. *J. Biol. Chem.* **288**, 16949–16959 (2013). doi:10.1074/jbc.M112.420521 Medline
27. R. Fernandez-Leiro, J. Conrad, J.-C. Yang, S. M. V. Freund, S. H. W. Scheres, M. H. Lamers, Self-correcting mismatches during high-fidelity DNA replication. *Nat. Struct. Mol. Biol.* **24**, 140–143 (2017). doi:10.1038/nsmb.3348 Medline
28. L. S. Beese, V. Derbyshire, T. A. Steitz, Structure of DNA polymerase I Klenow fragment bound to duplex DNA. *Science* **260**, 352–355 (1993). doi:10.1126/science.8469987 Medline
29. B. Malone, J. Chen, Q. Wang, E. Llewellyn, Y. J. Choi, P. D. B. Olinares, X. Cao, C. Hernandez, E. T. Eng, B. T. Chait, D. E. Shaw, R. Landick, S. A. Darst, E. A. Campbell, Structural basis for backtracking by the SARS-CoV-2 replication-transcription complex. *Proc. Natl. Acad. Sci. U.S.A.* **118**, e2102516118 (2021). doi:10.1073/pnas.2102516118 Medline
30. S. Jockusch, C. Tao, X. Li, M. Chien, S. Kumar, I. Morozova, S. Kalachikov, J. J. Russo, J. Ju, Sofosbuvir terminated RNA is more resistant to SARS-CoV-2 proofreader than RNA terminated by Remdesivir. *Sci. Rep.* **10**, 16577 (2020). doi:10.1038/s41598-020-73641-9 Medline
31. M. L. Agostini, E. L. Andres, A. C. Sims, R. L. Graham, T. P. Sheahan, X. Lu, E. C. Smith, J. B. Case, J. Y. Feng, R. Jordan, A. S. Ray, T. Cihlar, D. Siegel, R. L. Mackman, M. O. Clarke, R. S. Baric, M. R. Denison, Coronavirus Susceptibility to the Antiviral Remdesivir (GS-5734) Is Mediated by the Viral Polymerase and the Proofreading Exoribonuclease. *mBio* **9**, e00221-18 (2018). doi:10.1128/mBio.00221-18 Medline
32. J. Shim, G. Larson, V. Lai, S. Naim, J. Z. Wu, Canonical 3′-deoxyribonucleotides as a chain terminator for HCV NS5B RNA-dependent RNA polymerase. *Antiviral Res.* **58**, 243–251 (2003). doi:10.1016/S0166-3542(03)00007-X Medline
33. G. Campagnola, P. Gong, O. B. Peersen, High-throughput screening identification of poliovirus RNA-dependent RNA polymerase inhibitors. *Antiviral Res.* **91**, 241–251 (2011). doi:10.1016/j.antiviral.2011.06.006 Medline

34. C. Kao, M. Zheng, S. Rüdisser, A simple and efficient method to reduce nontemplated nucleotide addition at the 3 terminus of RNAs transcribed by T7 RNA polymerase. *RNA* **5**, 1268–1272 (1999). doi:10.1017/S1355838299991033 Medline
35. S. Q. Zheng, E. Palovcak, J.-P. Armache, K. A. Verba, Y. Cheng, D. A. Agard, MotionCor2: Anisotropic correction of beam-induced motion for improved cryo-electron microscopy. *Nat. Methods* **14**, 331–332 (2017). doi:10.1038/nmeth.4193 Medline
36. J. Zivanov, T. Nakane, S. H. W. Scheres, Estimation of high-order aberrations and anisotropic magnification from cryo-EM data sets in *RELION*-3.1. *IUCrJ* **7**, 253–267 (2020). doi:10.1107/S2052252520000081 Medline
37. A. Punjani, J. L. Rubinstein, D. J. Fleet, M. A. Brubaker, cryoSPARC: Algorithms for rapid unsupervised cryo-EM structure determination. *Nat. Methods* **14**, 290–296 (2017). doi:10.1038/nmeth.4169 Medline
38. A. Punjani, H. Zhang, D. J. Fleet, Non-uniform refinement: Adaptive regularization improves single-particle cryo-EM reconstruction. *Nat. Methods* **17**, 1214–1221 (2020). doi:10.1038/s41592-020-00990-8 Medline
39. T. D. Goddard, C. C. Huang, E. C. Meng, E. F. Pettersen, G. S. Couch, J. H. Morris, T. E. Ferrin, UCSF ChimeraX: Meeting modern challenges in visualization and analysis. *Protein Sci.* **27**, 14–25 (2018). doi:10.1002/pro.3235 Medline
40. Y. Z. Tan, P. R. Baldwin, J. H. Davis, J. R. Williamson, C. S. Potter, B. Carragher, D. Lyumkis, Addressing preferred specimen orientation in single-particle cryo-EM through tilting. *Nat. Methods* **14**, 793–796 (2017). doi:10.1038/nmeth.4347 Medline
41. A. Casañal, B. Lohkamp, P. Emsley, Current developments in Coot for macromolecular model building of Electron Cryo-microscopy and Crystallographic Data. *Protein Sci.* **29**, 1069–1078 (2020). doi:10.1002/pro.3791 Medline
42. J. A. Pleiss, M. L. Derrick, O. C. Uhlenbeck, T7 RNA polymerase produces 5′ end heterogeneity during in vitro transcription from certain templates. *RNA* **4**, 1313–1317 (1998). doi:10.1017/S135583829800106X Medline
43. P. V. Afonine, B. K. Poon, R. J. Read, O. V. Sobolev, T. C. Terwilliger, A. Urzhumtsev, P. D. Adams, Real-space refinement in PHENIX for cryo-EM and crystallography. *Acta Crystallogr. D* **74**, 531–544 (2018). doi:10.1107/S2059798318006551 Medline
44. V. B. Chen, W. B. Arendall 3rd, J. J. Headd, D. A. Keedy, R. M. Immormino, G. J. Kapral, L. W. Murray, J. S. Richardson, D. C. Richardson, MolProbity: All-atom structure validation for macromolecular crystallography. *Acta Crystallogr. D* **66**, 12–21 (2010). doi:10.1107/S0907444909042073 Medline
45. F. Madeira, Y. M. Park, J. Lee, N. Buso, T. Gur, N. Madhusoodanan, P. Basutkar, A. R. N. Tivey, S. C. Potter, R. D. Finn, R. Lopez, The EMBL-EBI search and sequence analysis tools APIs in 2019. *Nucleic Acids Res.* **47**, W636–W641 (2019). doi:10.1093/nar/gkz268 Medline
46. X. Robert, P. Gouet, Deciphering key features in protein structures with the new ENDscript server. *Nucleic Acids Res.* **42**, W320–W324 (2014). doi:10.1093/nar/gku316 Medline
47. C. A. Schneider, W. S. Rasband, K. W. Eliceiri, NIH Image to ImageJ: 25 years of image analysis. *Nat. Methods* **9**, 671–675 (2012). doi:10.1038/nmeth.2089 Medline

ACKNOWLEDGMENTS

We thank S. Wu for help with cryo-EM data collection at Yale Titan Krios cryo-EM facility. We particularly thank Dr. Ronald Breaker at Yale University for his support during the early phases of this work. **Funding:** This work was supported by NVIDIA GPU Grant Program (C.L.), the start-up funding granted to B.L. from the Hormel Institute, University of Minnesota and the start-up funding granted to Y.Y. from Iowa State University. **Author contributions:** Y.Y. and C.L. conceived and designed the experiments. Y.Y. performed the biochemical characterizations. Y.Y., B.L, and W.S. prepared the cryo-EM samples and collected cryo-EM data. Y.Y., C.L. and B.L. processed the cryo-EM data. C.L. and Y.Y. performed modeling building and structural analyses. Y.Y. and C.L. wrote the manuscript with input from S.T.B., D.G.S and B.L. **Competing interests:** The authors declare no competing interests. **Data and materials availability:** Atomic coordinates of three structures determined in this study have been deposited in PDB with accession codes 7N0B (SARS-CoV-2 nsp10-nsp14 (WT)-RNA complex), 7N0C (SARS-CoV-2 nsp10-nsp14 (E191A)-RNA complex, monomer) and 7N0D (SARS-CoV-2 nsp10-nsp14 (E191A)-RNA complex, tetramer). The cryo-EM maps have been deposited in the Electron Microscopy Data Bank with accession number EMD-24102 (SARS-CoV-2 nsp10-nsp14 (WT)-RNA complex), EMD-24103 (SARS-CoV-2 nsp10-nsp14 (E191A)-RNA complex, monomer) and EMD-24104 (SARS-CoV-2 nsp10-nsp14 (E191A)-RNA complex, tetramer). Materials are available from Y.Y. upon request and may require a material transfer agreement. This work is licensed under a Creative Commons Attribution 4.0 International (CC BY 4.0) license, which permits unrestricted use, distribution, and reproduction in any medium, provided the original work is properly cited. To view a copy of this license, visit https://creativecommons.org/licenses/by/4.0/. This license does not apply to figures/photos/artwork or other content included in the article that is credited to a third party; obtain authorization from the rights holder before using such material.

SUPPLEMENTARY MATERIALS

science.sciencemag.org/cgi/content/full/science.abi9310/DC1
Materials and Methods
Figs. S1 to S9
Table S1
References (*34*–*47*)
MDAR Reproducibility Checklist

9 April 2021; accepted 20 July 2021
Published online 27 July 2021
10.1126/science.abi9310

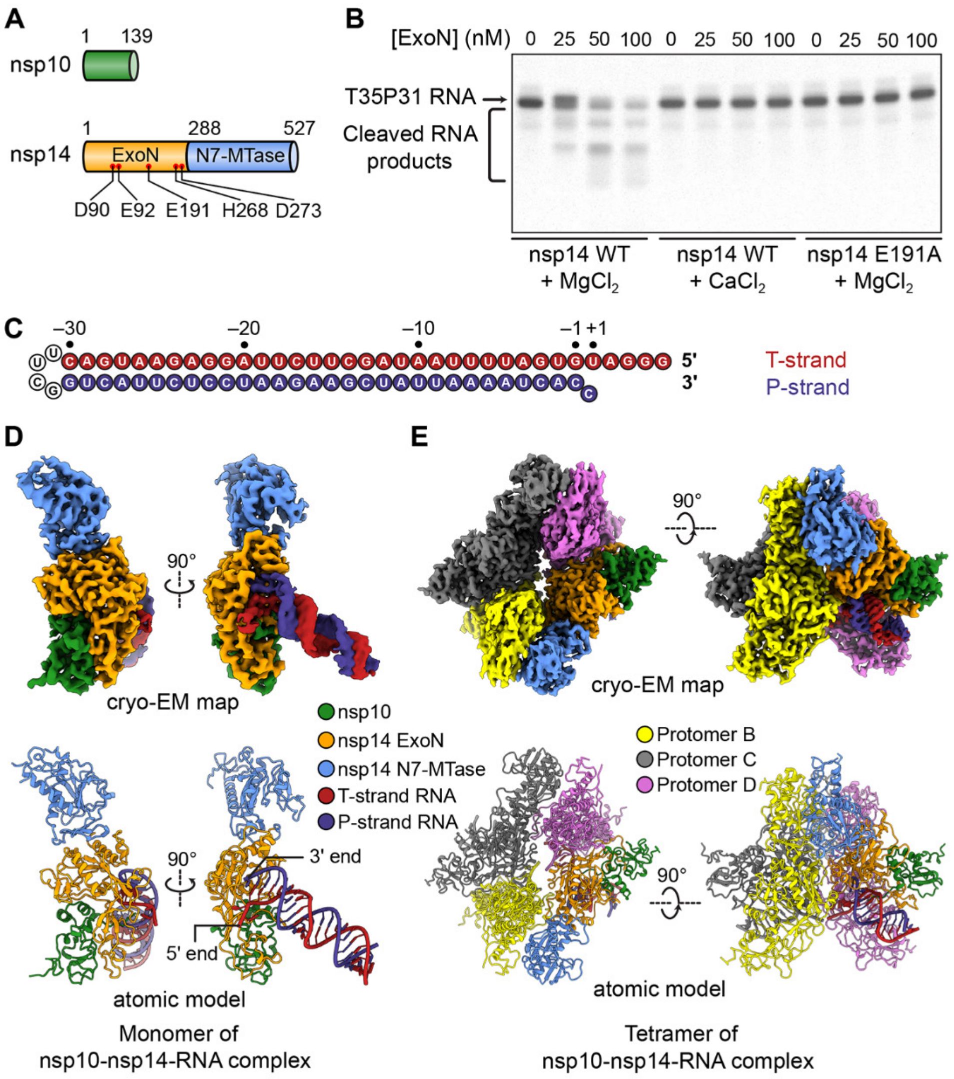

Fig. 1. Structural and functional overview of SARS-CoV-2 nsp10-nsp14-RNA complexes. (**A**) Domain organization of SARS-CoV-2 nsp10 and nsp14. Domain boundary residues are numbered. Five catalytic residues in the nsp14 ExoN domain are indicated as red dots and are labeled. (**B**) Cleavage of T35P31 RNA substrate by SARS-CoV-2 nsp10-nsp14 ExoN complex. The concentrations (in nM) of wild-type (WT) or E191A mutant ExoN are indicated. The RNAs were resolved by denaturing polyacrylamide gel electrophoresis (PAGE) and stained by SYBR Gold. A representative result from three biological replicates is shown. (**C**) Sequence and numbering of the T35P31 RNA substrate used in biochemical characterization and structural determination. T-strand, template strand; P-strand, product strand. T-strand and P-strand RNAs are connected by a UUCG tetraloop. (**D**) Cryo-EM map and atomic model of the monomeric form of SARS-CoV-2 nsp10-nsp14-RNA complex. (**E**) Cryo-EM map and atomic model of the tetrameric form of SARS-CoV-2 nsp10-nsp14-RNA complex.

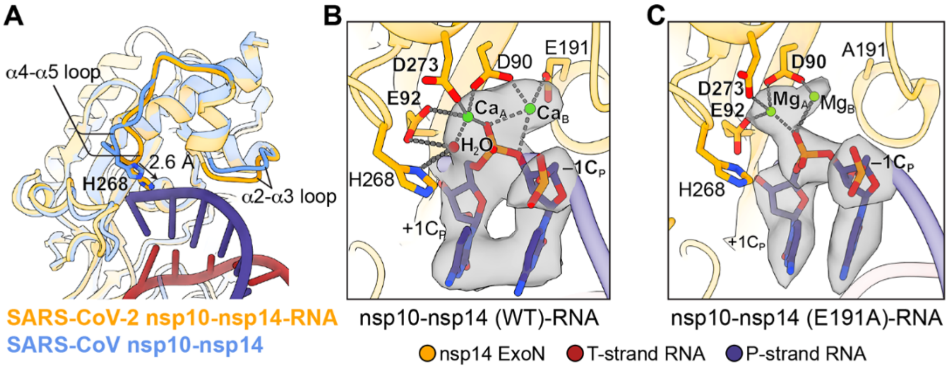

Fig. 2. Active site conformation and catalytic mechanism of SARS-CoV-2 ExoN. (**A**) Superimposition of SARS-CoV nsp10-nsp14 complex (cornflower blue, PDB 5C8U) and SARS-CoV-2 nsp10-nsp14-RNA complex (orange) illustrates the conformational changes of α2-α3 and α4-α5 loops and a 2.6 Å shift of H268 toward the RNA upon substrate binding. (**B**) Active site structure of SARS-CoV-2 nsp10-nsp14 (WT)-RNA complex. Ca^{2+} ions, green spheres; catalytic water, red sphere. Nucleotide residues in P-strand RNA are indicated with subscript "P." $+1C_P$, $-1C_P$, the catalytic water and two active site metal ions are superimposed with their cryo-EM densities contoured at 10σ. (**C**) Active site structure of SARS-CoV-2 nsp10-nsp14 (E191A)-RNA complex. Mg^{2+} ions, green spheres. $+1C_P$, $-1C_P$, and two active site metal ions are superimposed with their cryo-EM densities contoured at 7σ.

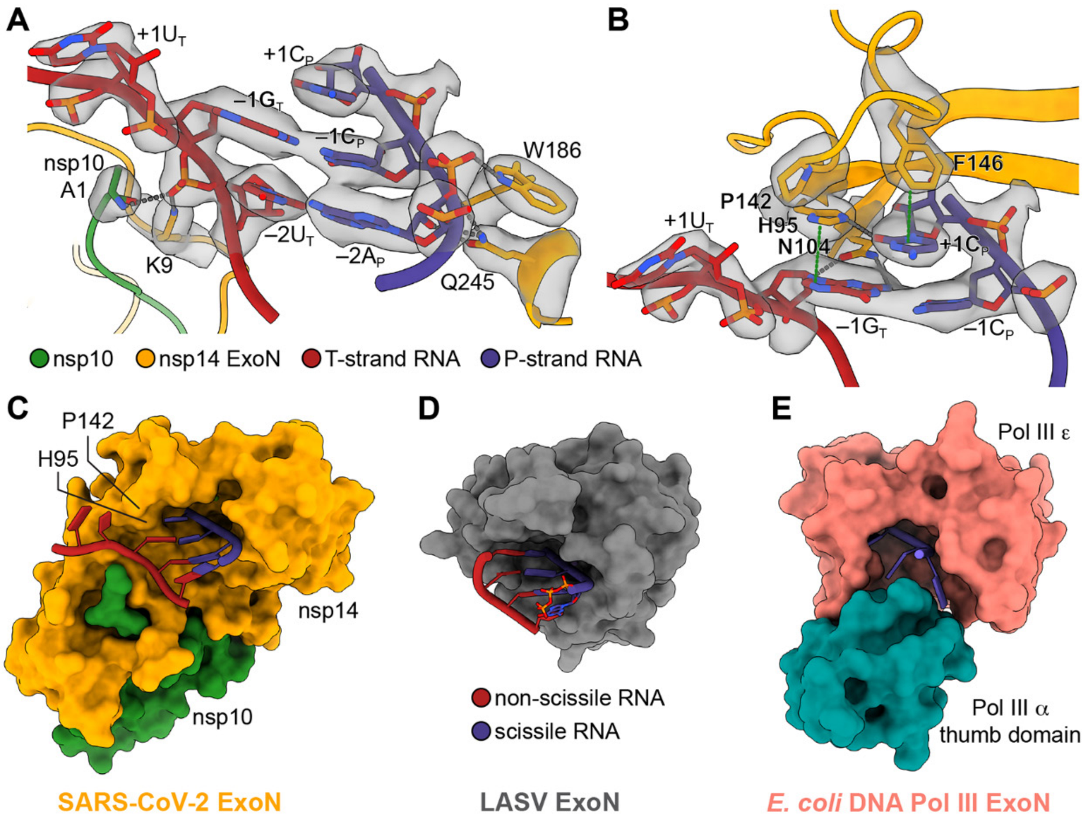

Fig. 3. Mechanism of substrate recognition by SARS-CoV-2 ExoN. (**A**) Interactions between SARS-CoV-2 nsp10-nsp14 ExoN and T35P31 RNA backbone. Nucleotide residues in T-strand RNA are indicated with subscript "T," nucleotide residues in P-strand RNA are indicated with subscript "P." Hydrogen bonds and salt bridges are shown as gray dotted lines. Interacting nucleotide and protein residues are superimposed with their cryo-EM densities contoured at 7σ. (**B**) Interactions between SARS-CoV-2 nsp10-nsp14 ExoN and T35P31 RNA at +1 and −1 nucleobases positions. Hydrogen bonds and salt bridges are shown as gray dotted lines. π-π stacking interactions are indicated by green dotted lines. Interacting nucleotide and protein residues are superimposed with their cryo-EM densities contoured at 7σ. (**C**) Surface representation of SARS-CoV-2 nsp10-nsp14 ExoN substrate-binding pocket shows a restricted opening on the T-strand side that prevents base-pairing at substrate RNA +1 position. For clarity, the N7-MTase domain of nsp14 is not shown. (**D**) Surface representation of LASV NP ExoN domain. A fully base-paired dsRNA substrate (shown as cartoons) is bound in the substrate-binding pocket of LASV ExoN. (**E**) Surface representation of *E. coli* DNA Pol III ExoN complex. The narrow substrate-binding pocket allows the entry of ssDNA only.

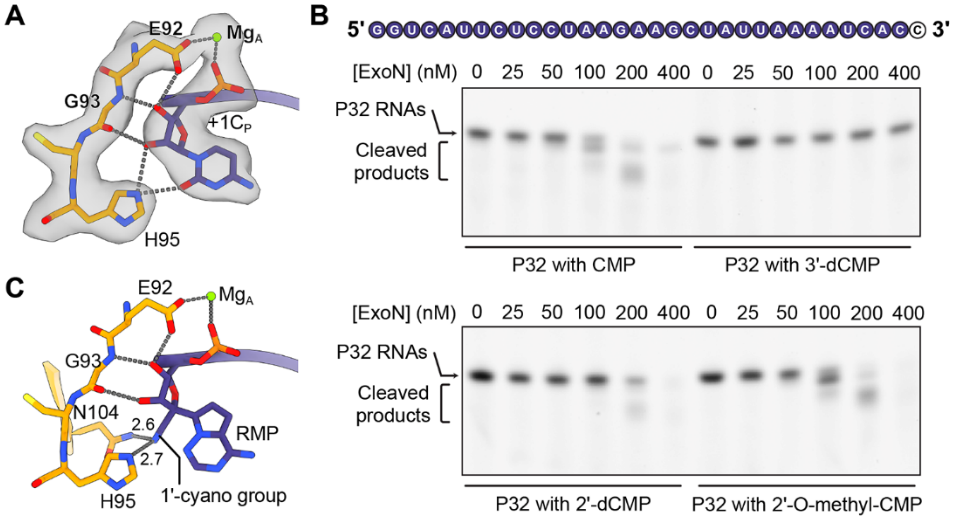

Fig. 4. Structural insights into antiviral design. (A) Interactions between SARS-CoV-2 nsp10-nsp14 ExoN and 3′-end nucleotide mediated by its 2′- and 3′-OH groups. Mg^{2+} ion, green sphere. Hydrogen bonds are shown as gray dotted lines. $+1C_P$ and its interacting protein residues are superimposed with cryo-EM densities contoured at 7σ. (B) Cleavage of various P32 ssRNA substrates by SARS-CoV-2 nsp10-nsp14 complex. The 3′-end CMP that bears different modifications at its 2′- or 3′-OH groups is shown as a black letter in white circles. The concentrations (in nM) of ExoN are indicated. The RNAs were resolved by denaturing polyacrylamide gel electrophoresis (PAGE) and stained by SYBR Gold. A representative result from three biological replicates is shown. (C) Predicted interactions between SARS-CoV-2 nsp10-nsp14 ExoN and remdesivir monophosphate (RMP), which is modeled at the 3′-end +1 position of P-strand RNA.
